# Molecular pathways affecting reproductive efficiency in seasonal breeders: prospects and implications for improving fertility in donkeys

**DOI:** 10.3389/fvets.2025.1633945

**Published:** 2025-10-15

**Authors:** Muhammad Faheem Akhtar, Shahzad Ali, Faizul Hassan, Wang Changfa

**Affiliations:** ^1^Liaocheng Research Institute of Donkey High-Efficiency Breeding and Ecological Feeding, Liaocheng University, Liaocheng, China; ^2^Department of Breeding and Genetics, Cholistan University of Veterinary and Animal Sciences, Bahawalpur, Pakistan

**Keywords:** seasonal breeding, donkeys, hypothalamic–pituitary-gonadal axis, melatonin, KNDy neurons, oxidative stress, steroidogenesis, reproductive efficiency

## Abstract

Intense neuroendocrine and molecular pathways with environmental sensitivity maintain reproductive efficiency in seasonal breeders, together with donkeys. The hypothalamic–pituitary-gonadal (HPG) axis functions as a primary controller through modifying gonadotropin-releasing hormone (GnRH) secretion that depends on melatonin levels, which induces photoperiodic instructions to the system. The activation of HPG axis is triggered by decreasing melatonin levels during long-day seasons, yet sustained high levels of melatonin during short-day seasons cause its suppression. The reproductive pulsatility of GnRH depends on kisspeptin-neurokinin B-dynorphin (KNDy) neurons, which are controlled by melatonin through activity regulation to produce seasonal reproductive suppression. Reproductive ability depends on metabolic signaling, which connects nutrient availability to gonadal functions to maintain fertility during optimum nutritional status. Studies have demonstrated that oxidative stress is a primary disruptor of reproductive functions as it produces gonadal cell damage while stopping steroid synthesis and increasing cell death. Endocrine-disrupting chemicals (EDCs) cause additional reproductive problems through interfering with steroidogenic enzymes, which results in hormonal imbalance and infertility. Prolactin works in association with gonadotropins and metabolic pathways to control reproductive adaptations under seasonal variation. Understanding of molecular mechanisms is essential for increasing reproductive success among donkeys and other seasonal breeders in general. The breeding programs might benefit from solutions such as photoperiod manipulation and melatonin treatments, together with nutritional supplementation and antioxidant therapies. The review focuses on seasonal reproductive processes, endocrinology, assisted reproductive technologies (ARTs), and peculiarities of anatomy and behavior. Discoveries in sperm vitrification, testicular immunology, metabolic endocrinology, and follicular dynamics give important clues to fertility manipulation in this species and suggest interventions to be pursued to enhance fertility outcomes and conservation approaches.

## Introduction

Donkeys are long-lived polyestrous equids that are vital to livelihoods and biodiversity. The reproductive efficiency of farm mammals, including donkeys, is affected by seasonal variations, which subsequently impacts production traits by affecting milk yield together with meat quality and reproductive outcomes (1). Some mammals such as Cattle exhibit regular reproductive cyclicity throughout the year, yet sheep, together with goats, horses, and donkeys, demonstrate seasonal breeding cycles with peak births in late stages and early spring to maximize offspring survival (2). The reproduction cycles of these species function through neuroendocrine systems that control the frequency of ovulation along with spermatogenic activity, gamete quality, and sexual behavior (3). The regulation of seasonal reproduction depends on two main factors: natural endogenous circannual rhythms and external photoperiod signals, which the pineal gland, mediating melatonin secretion, controls (4). External signals adjust hypothalamic–pituitary-gonadal (HPG) axis functioning so the reproductive cycles undergo major neuroendocrine alterations (5). Although donkeys are distributed worldwide, the reproductive inefficiency of reproduction, especially due to seasonality and metabolic-endocrine interactions, restricts their productivity and conservation (6).

Artificial breeding programs use photoperiodic manipulation through external daylight exposure for mares, sheep, and goats, together with melatonin supplementation specifically for sheep and goats to achieve seasonal reproductive synchronization as well as seasonal reproductive control (7). The interventions alongside genetic selection programs focus on maximizing reproductive performance within controlled breeding programs (8).

Donkeys are long-day breeders as the estrous cycle is more regular and pronounced during long-day periods (9). The reproductive activity peaks in spring and summer, while it is reduced or exhibits anestrus in autumn and winter (10). Almost every reproductive parameter of donkeys varies with seasonal variability, like foaling rate, which is higher in warmer months due to increased mating success and favorable conditions, improved semen motility and concentration during spring and early summer (11).

Donkey reproductive patterns respond to various molecular systems that combine hormones with energy homeostasis and natural environmental stimuli, including light duration, weather, and diet quality (9, 11). Unlike horses, donkeys have a distinctive reproductive physiology that requires species-specific investigations and molecular treatments (12). The purpose of this review is to summarize what is known about donkey sexual biology and suggest biologically realistic ways to improve fertility.

## Hypothalamic–pituitary-gonadal axis dysregulation

The hypothalamic–pituitary-gonadal (HPG) axis functions as the primary mechanism to regulate reproductive functions among all mammals, including the donkey, although it operates as a seasonal breeder (11). Through this axis, the hypothalamus produces gonadotropin-releasing hormone (GnRH) in pulsatile patterns that trigger the anterior pituitary to release both luteinizing hormone (LH) and follicle-stimulating hormone (FSH) (13). The gonadotropins exercise their effects on the gonads to control gametogenesis as well as hormone synthesis, where males produce testosterone and females produce estrogen and progesterone (14).

### Seasonal regulation of the HPG axis

Seasonal breeders closely link their reproductive efficiency to environmental cues that mainly include photoperiod (day length), temperature, and nutritional status (15). The pineal gland produces melatonin as a response to dark conditions, which controls the seasonal pattern of GnRH secretion (16). From winter months’ short-day periods, melatonin secretion extends over time until it suppresses GnRH release, which reduces LH and FSH secretion and causes reproductive inactivity (17). The reduction of melatonin during long-day periods results in the reactivation of GnRH pulsatile action and restores reproductive capacity (18).

Relational dynamics of the HPG axis are most prominent in mares together with sheep, goats, and donkeys because their breeding patterns match photoperiod modifications of melatonin release (16). Donkeys share the reproductive pattern of horses by being long-day breeders, and their breeding season occurs during spring and summer when day length expands (11). The natural birth cycle results in foal births when environmental conditions offer the best resources.

### Stress-induced dysregulation of the HPG axis

The hypothalamic–pituitary–adrenal (HPA) axis that controls stress responses creates a feedback mechanism with the HPG axis. Between chronic stress and HPA axis activation arises the production of corticotropin-releasing hormone (CRH) and adrenocorticotropic hormone (ACTH) that stimulate cortisol production from adrenal glands (19). The release of GnRH diminishes when cortisol levels increase, which subsequently decreases LH and FSH production, thus leading to reproductive system suppression.

The release of GnRH decreases when cortisol levels increase, which subsequently reduces LH and FSH production, thereby leading to suppression of the reproductive system (20). The reproductive system of female seasonal breeders shows adverse effects from chronic stress because this results in anovulation together with irregular estrous cycles and reduced estrogen production, which frequently causes ovarian dysfunction and persistent follicles or ovarian cysts (21). The prolonged exposure to stress in male individuals decreases testosterone levels along with spermatogenesis and causes sperm quality to decline while diminishing sexual desire, so fertility remains impaired in breeding periods (22).

The neurochemical agents serotonin (5-HT), along with dopamine and norepinephrine (NE), function as vital elements for controlling HPG axis responses under stress conditions (23). GnRH release receives stimulation from serotonin, although the changing sensitivity of serotonin receptors during seasonal periods may contribute to reproductive suppression caused by environmental stressors like nutritional deficiencies, changes in social standing, and climate patterns (24).

### Metabolic and nutritional effects on the HPG axis

The HPG axis operates under significant control from energy balance regulation. Reproductive function regulation occurs through the AMPK-mTOR signaling pathway that detects energy levels by influencing GnRH neurons (19). AMPK activation stops GnRH secretion to cause reproductive dormancy when the body faces nutritional hardships or negative energy conditions (such as winter months). The HPG axis receives a signal from adequate energy storage to activate mTOR signaling, which then triggers GnRH release and increases reproductive capability (25).

**Figure 1:**
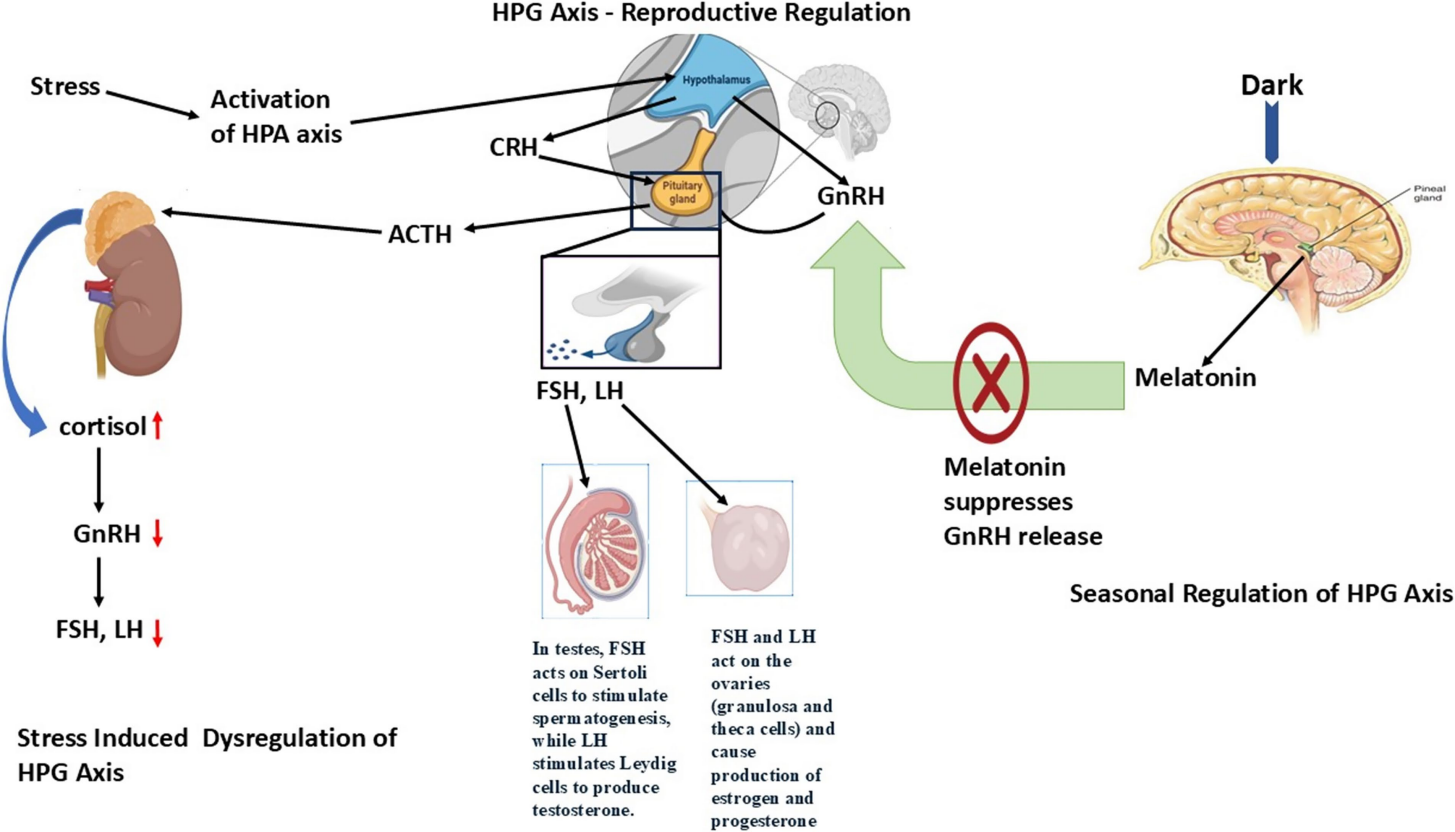
Role of hypothalamic–pituitary-gonadal (HPG) Axis dysregulation in regulating reproduction in seasonal breeders. The HPG axis is a hormonal system involving the hypothalamus, pituitary gland, and gonads that controls reproduction through GnRH, LH/FSH, and sex steroids. Dysregulation occurs due to stress, malnutrition, endocrine disruptors, or seasonal changes, leading to impaired hormone secretion, disrupted gametogenesis, and reduced fertility.

The reproductive hormone regulation of donkeys that experience seasonality depends on their body condition changes and how much they eat between seasons (26). Studies performed on mares and sheep proved that minimal body fat, together with low leptin levels, restrict normal GnRH signal pulsing which causes breeding season delays. The HPG axis becomes fully active once the nutritional condition improves, thus breeding occurs at the most appropriate time for the environment (27). The molecular pathway Hypothalamic–Pituitary-Gonadal (HPG) Axis Dysregulation is shown in Figure 1.

There is a different gonadotropin secretion pattern in donkeys. Jennies exhibit two FSH peaks during one estrous cycle and a long-lasting LH surge that frequently continues after ovulation (28). This differs from the single peak of FSH and closely timed LH surge in mares and ewes. These hormonal patterns can provide distinct follicular and luteal sustainability processes in donkeys (29).

An impressive molecular difference is seen in the ligand specificity of the FSH receptor (FSHR). Cloned FSHRs in donkeys can to bind FSH and LH/chorionic gonadotropin (CG) in a ligand promiscuous manner, which has not been observed in horses or sheep (30, 31). This is due to differences in amino acids of the extracellular domain of the receptor (~96% homology with equine FSHR). The physiological significance of such receptor flexibility on follicular development has yet to be understood (32).

### LH and eCG bioactivity

Equine chorionic gonadotropin (eCG) is LH-like, as well as FSH-like, in non-equines (33). Nevertheless, in donkeys, LH and CG are largely LH-active with little FSH-like activity in *in vitro* assays. This also emphasizes species-specific hormone-receptor interactions that may have effects on ovulatory regulation and folliculogenesis (29).

### Implications of HPG axis for seasonal breeding management

The mechanism through the HPG axis functions in seasonal breeders complicates the management of reproduction and breeding operations. The following potential strategies can be used to improve reproductive efficiency in seasonal breeders (34):

1. Artificial lighting techniques that replicate long-day conditions can be employed to induce early estrus in mares and donkeys, thus increasing their reproductive effectiveness.
2. The body condition managed properly during the pre-breeding period reduces the impact of seasonal reproductive suppression.
3. The outcome of fertility improves when stress levels decrease through reducing environmental and social pressure factors.

The reproductive system of donkeys, alongside other seasonal breeders, controls the HPG axis through interactions between photoperiod signals, metabolic indicators, and stress responses (35). The reproductive efficiency of animals becomes compromised when the stress axis becomes nonfunctioning due to chronic stress combined with poor nutritional status, along with unsuitable environmental elements (36). Understanding seasonal regulatory processes better will allow scientists to create specific measures that enhance the breeding performance of seasonal species. The summary of the molecular pathway is shown in Table 1.

**Table 1:** HPG Axis dysregulation in seasonal breeders.

Factor	Potential effects on reproductive functions
Role of HPG Axis dysregulation	Disrupts GnRH, LH, and FSH release, thus impairs follicular development, ovulation, or spermatogenesis, reducing reproductive efficiency (22).
Seasonal regulation	Photoperiod influences melatonin secretion, which modulates GnRH pulsatility, leading to reproductive activation in long-day breeders (166).
Photoperiodic influence	Long days → Reduced melatonin → Increased GnRH → Reproductive activation. Short days → Increased melatonin → Suppressed GnRH → Reproductive inactivity (167).
Stress-induced dysregulation	Chronic stress activates the HPA axis, increasing cortisol levels, which suppresses GnRH, leading to reproductive dysfunction (anovulation, poor sperm quality) (22).
Neurochemical modulation	Serotonin stimulates GnRH; dopamine and norepinephrine contribute to seasonal reproductive regulation. Environmental stressors impact neurotransmitter sensitivity (168).
Management strategies	Artificial lighting to induce estrus, optimized nutrition to prevent reproductive suppression, and stress reduction to improve breeding outcomes (21).

## Melatonin signaling pathway (photoperiodic regulation)

In seasonal breeders, the information on photoperiod is encoded in the melatonin secretion of the pineal gland, which regulates the hypothalamic–pituitary-gonadal (HPG) axis (12). In sheep, melatonin can influence the pituitary pars tuberalis MT1 and MT2 receptors in the pars tuberalis to modulate TSH and downstream thyroid hormones, which then modulate kisspeptin and GnRH release (37). It is the same with horses, which express melatonin receptors in the hypothalamus, pituitary, and ovary (38).

Comparatively, there is no information on the expression and signaling of melatonin receptors in donkeys. The submissive photoperiodic reactivity of the species and the lack of clear seasonal patterns indicate that melatonin transduction may be distorted or an alternative environmental signaling may be used (39).

### Role of photoperiod in seasonal reproduction

The reproductive patterns of seasonal breeders are controlled through environmental signals that primarily include changes in day length (photoperiod) (40). The adaptation brings about offspring births in optimal times, which usually matches the spring season when environmental factors create favorable conditions for survival. The main regulator for this process functions through melatonin, which the pineal gland produces because of daily darkness. Through its neuroendocrine role, melatonin carries photoperiodic data, which subsequently affects reproductive hormone release (41).

Horses, together with donkeys, show suppressed melatonin production when daylight stretches out, which activates their gonads (42). Short-day breeders like sheep and goats activate reproduction during periods when their nightly melatonin hormone production reaches higher levels. Knowledge of the melatonin signaling pathway stands vital for determining the seasonal mechanisms thatdonkey reproductive effectiveness (43).

### Mechanism of mammalian seasonal reproduction

#### Light perception and transmission to the pineal gland

Relying on the retina for light detection stands as the main photoreceptor mechanism for mammals since birds use deep-brain photoreceptors (44). The ipRGCs inside the retina carry melanopsin photopigment as they detect light exposure through their intrinsic photosensitive function. The photic signals travel through the retinohypothalamic tract (RHT) until they reach the suprachiasmatic nucleus (SCN) of the hypothalamus, which acts as the central circadian pacemaker (45). Light information from the SCN passes through the PVN and IML section of the spinal cord before reaching the SCG, which makes its way to the pineal gland (46).

When light stimulation ends in darkness, the SCN exhibits reduced activity, while norepinephrine (NE) produced in the SCG activates *β*-adrenergic receptors in pinealocytes through these receptors. The successive neural events increase the activity of arylalkylamine N-acetyltransferase (AANAT), which results in nighttime melatonin production (47).

#### Melatonin as a photoperiodic messenger

The secretion of melatonin follows a daily cycle of 24 h, where the hormone remains in the body for an amount equivalent to the duration. It connects to MT1 and MT2 melatonin receptors, which exist mainly inside the pars tuberalis (PT) from the pituitary gland as well as the hypothalamus (48). The MT1 receptor functions as the primary photoperiodic information transmitter within seasonal breeders because it displays high expression levels in these animals (49).

The secretion of melatonin decreases in donkeys and horses when the photoperiod lasts longer, which activates the hypothalamic–pituitary-gonadal (HPG) axis to increase gonadotropin-releasing hormone (GnRH) release (50). The regulatory hormone melatonin creates positive effects on gonadal activity through thyroid hormone regulation of the mediobasal hypothalamus (MBH) during extended exposure durations in short-day breeders like sheep and goats (51).

#### Thyroid hormone regulation in seasonal breeders

The regulation of seasonal reproduction by melatonin occurs mainly through modifications in thyroid hormone metabolic patterns. The posterior pituitary (PT), a region of the pituitary gland, plays a crucial role in this process (52).

The reduction of melatonin activates thyroid-stimulating hormone (TSHβ) expression in the PT under long-day stimulus conditions. The activity of type 2 deiodinase (DIO2) is downregulated, resulting in the hypothalamus producing less triiodothyronine (T3). The stimulation of the reproductive axis occurs because of this process, and it advances both follicular development and spermatogenesis (53).

The extended exposure to melatonin stimulates TSHβ expression in the PT, which activates DIO3 to convert T3 into inactive reverse T3 through its enzymatic activity. The hormone GnRH becomes suppressed, which prevents the release of reproductive signals during seasonal anestrous periods (54).

## Molecular mechanisms involved in melatonin signaling

### Role of circadian clock genes

The SCN acts as a circadian oscillator that manages melatonin production by controlling the expression of BMAL1 and CLOCK together with Period (Per1, Per2) and Cryptochrome (Cry1, Cry2). All these genes create a transcription-translation feedback loop that controls the length of melatonin production based on photoperiod (55).

Long-day conditions cause changes in the phase relationships of SCN neurons, which affect clock gene expression patterns and decrease melatonin production levels. The expression patterns of clock genes under short-day conditions extend melatonin production, that results in reproductive inhibition in donkeys long-day breeders (56).

Kisspeptin, originating from the Kiss1 gene, operates as a strong activator of GnRH release. The arcuate nucleus of the hypothalamus experiences decreased Kiss1 expression because of melatonin effects, which results in reproductive inactivity (57).

The reduction of melatonin levels in donkeys with long-day breeding patterns stimulates Kiss1 gene expression to trigger the activation of GnRH along with gonadotropins that initiate reproductive functions. Sheep display seasonal anestrus by having melatonin suppress Kiss1 expression, which prevents the release of GnRH (58).

### Role of RFamide-related peptides

The mammalian ortholog of gonadotropin-inhibitory hormone (GnIH) is RFamide-related peptide-3 (RFRP-3), which controls the activity of the HPG axis by suppression. The secretion of RFRP-3 shows both melatonin-regulated patterns and species-specific responses toward GnRH release (59). Sheep experience seasonal anestrus because RFRP-3 reduces GnRH secretion in their system. Short-day conditions stimulate RFRP-3 to boost GnRH secretion in the brains of hamsters, that helps the reproductive system to function normally (60). Researchers have not confirmed the function of RFRP-3 in donkeys, even though its relationship to Kisspeptin and thyroid hormone regulation might help understand seasonal reproductive patterns (61).

### Species-specific photoperiodic mechanisms

In sheep and horses, the melatonin TSH thyroid kisspeptin GnRH cascade regulates photoperiodic reproduction tightly (62). Donkeys might not be fully involved in this axis. Their reproduction physiology seems to be less responsive to a change of daylight, which suggests that they depend on other stimuli like dietary conditions, temperature, or socialization (63). This hypothesis has not been molecularly confirmed because there is a lack of neuroendocrine mapping.

### Applications in reproductive management of seasonal breeders

The examination of the melatonin signaling pathway enabled researchers to create methods that control reproductive patterns in donkeys along with other seasonal breeders (64).

**Figure 2:**
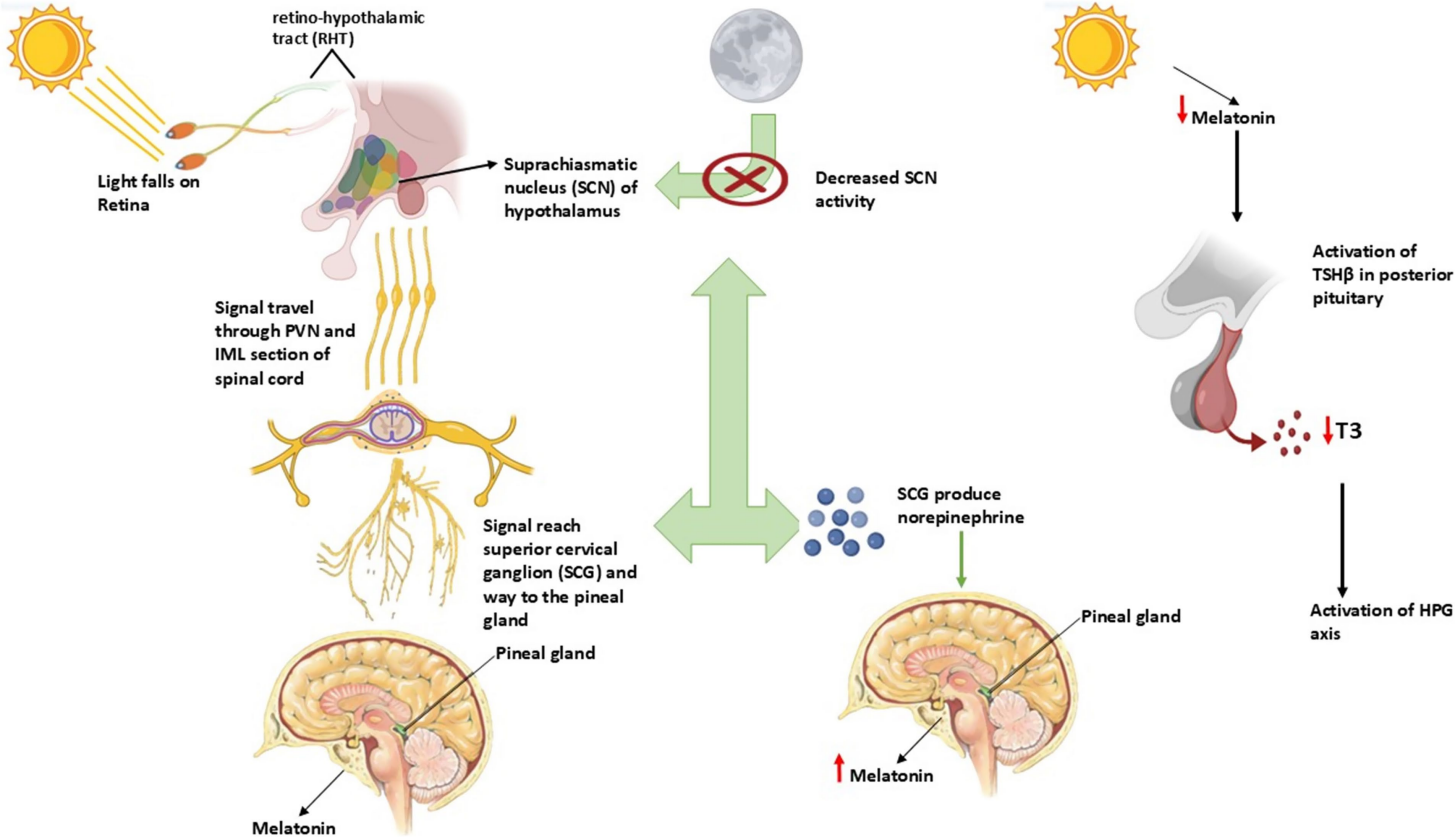
Role of photoperiod and its association with melatonin affecting reproduction in seasonal breeders.

1. Strategies involving artificial lighting can halt the production of melatonin, which leads to the acceleration of the breeding period. The technique is applied most frequently in equine breeding operations.
2. The administration of exogenous melatonin through implants provides a treatment that can halt reproduction while improving breeding seasons in animals with distinct seasonal cycle patterns.
3. Genetic Selection based on changing the photoperiodic responses has the potential to improve breeding outcomes of donkeys during unfavorable seasonal periods (Figure 2).

The reproductive efficiency of seasonal breeders, including donkeys heavily depends on the functioning of their melatonin signaling pathway. The neuroendocrine transducer function of melatonin depends on its ability to process photoperiodic cues with the SCN while working with PT and thyroid hormones alongside Kisspeptin and RFamide-related peptides (65). Knowledge advancement regarding these mechanisms enables developers to create successful breeding management strategies that improve reproductive success for donkeys and other seasonal breeders (66). The summary of the molecular pathway is shown in Table 2.

**Table 2:** Melatonin signaling pathway in seasonal breeders.

Aspect	Details
Role of photoperiod	Day length regulates seasonal reproduction through melatonin secretion, ensuring optimal timing for offspring birth (166).
Light perception	Retinal photoreceptors detect light → Signal relayed via Retin hypothalamic tract (RHT) → Suprachiasmatic nucleus (SCN) modulates melatonin production (167).
Melatonin secretion mechanism	Darkness triggers norepinephrine (NE) release → Activates pineal gland via *β*-adrenergic receptors → Stimulates AANAT enzyme → Increases melatonin synthesis (169).
Melatonin as a photoperiodic messenger	Melatonin binds to MT1 and MT2 receptors, influencing the hypothalamic–pituitary-gonadal (HPG) axis and seasonal reproductive cycles (48).
Effect on long-day breeders (donkeys, horses)	Longer daylight → Reduced melatonin → Increased GnRH → Activation of reproductive function (167).
Thyroid hormone regulation	Long-day exposure reduces melatonin → Increases TSHβ → Suppresses DIO3 → Elevates T3 → Activates reproductive function. Short-day exposure increases melatonin → Suppresses TSHβ → Activates DIO3 → Converts T3 into inactive form → Inhibits reproduction (170).
Circadian clock gene influence	SCN clock genes (BMAL1, CLOCK, Per, Cry) regulate melatonin secretion duration, affecting reproductive activation or suppression (171).
Kisspeptin’s association with melatonin	Melatonin inhibits Kiss1 gene expression in short-day conditions, reducing GnRH release and causing seasonal anestrus. In long-day breeders, reduced melatonin stimulates Kiss1 expression, triggering reproductive activity (172).

## Kisspeptin-neurokinin B-dynorphin neuron regulation

A major role in gonadotropin-releasing hormone (GnRH) secretion regulation belongs to the KNDy neuron system, which resides in the hypothalamic arcuate nucleus (ARC) (67). These neurons co-express three key neuropeptides: kisspeptin, neurokinin B (NKB), and dynorphin (Dyn). Kisspeptin functions as a powerful stimulant for GnRH release through the KNDy, neurons yet NKB activates the KNDy neurons at the same time Dyn plays an inhibitory role to maintain reproductive hormone balance (68).

Kisspeptin neurons play an important role in being upstream controllers of GnRH. Their expression is photoperiod-dependent and has been mapped in sheep and horses, where they gate seasonal activation of reproductive activity (69). The axis seems to be intact in donkeys: a kisspeptin analog (C6 peptide) can trigger ovulation and LH surges. The distribution, the density, and the photoperiodic control of kisspeptin neurons are however, still unknown, and this restricts us to comprehend its complete role in the reproductive physiology of the donkey (70, 71).

### Mechanism of KNDy neuron function

#### GnRH pulsatility regulation

The reproductive axis functions properly because GnRH secretion exists as pulsatile signals. The mechanical pulsations of neural signals depend on KNDy neurons through an auto-regulatory feedback mechanism (68). Neurokinin B (NKB) activates KNDy neurons through its stimulating effect, which produces more kisspeptin release. The direct activation of GnRH neurons by Kisspeptin results in elevated levels of GnRH hormone that is released into the bloodstream (72). The neurochemical activity of dynorphin creates negative feedback that limits KNDy neuron function for controlling GnRH release during required periods. The complex regulatory system maintains the correct timing of GnRH secretion because it functions as a crucial factor for reproductive health (73).

#### Experimental evidence of KNDy neuron function

- The GnRH pulse generator disappears permanently when scientists use NK3-SAP to destroy KNDy neurons, proving these cells hold the essential position for reproductive regulation (74).
- Studies involving ablating KNDy neurons establish that their destruction results in reduced gonadotropin release, infertility and body weight alterations (75).

#### KNDy neurons in seasonal breeders

- Donkeys belong to the seasonal breeders whose reproductive functions are controlled through seasonal photoperiod changes (11).
- The reduction of GnRH secretion and reproductive quiescence occurs because shorter daylight hours trigger melatonin secretion, which downregulates KNDy neuron activity (68).
- The reduction of melatonin secretion during breeding seasons enables KNDy neurons to become active once more, which results in fertility (76).

#### Additional regulatory factors

**Figure 3:**
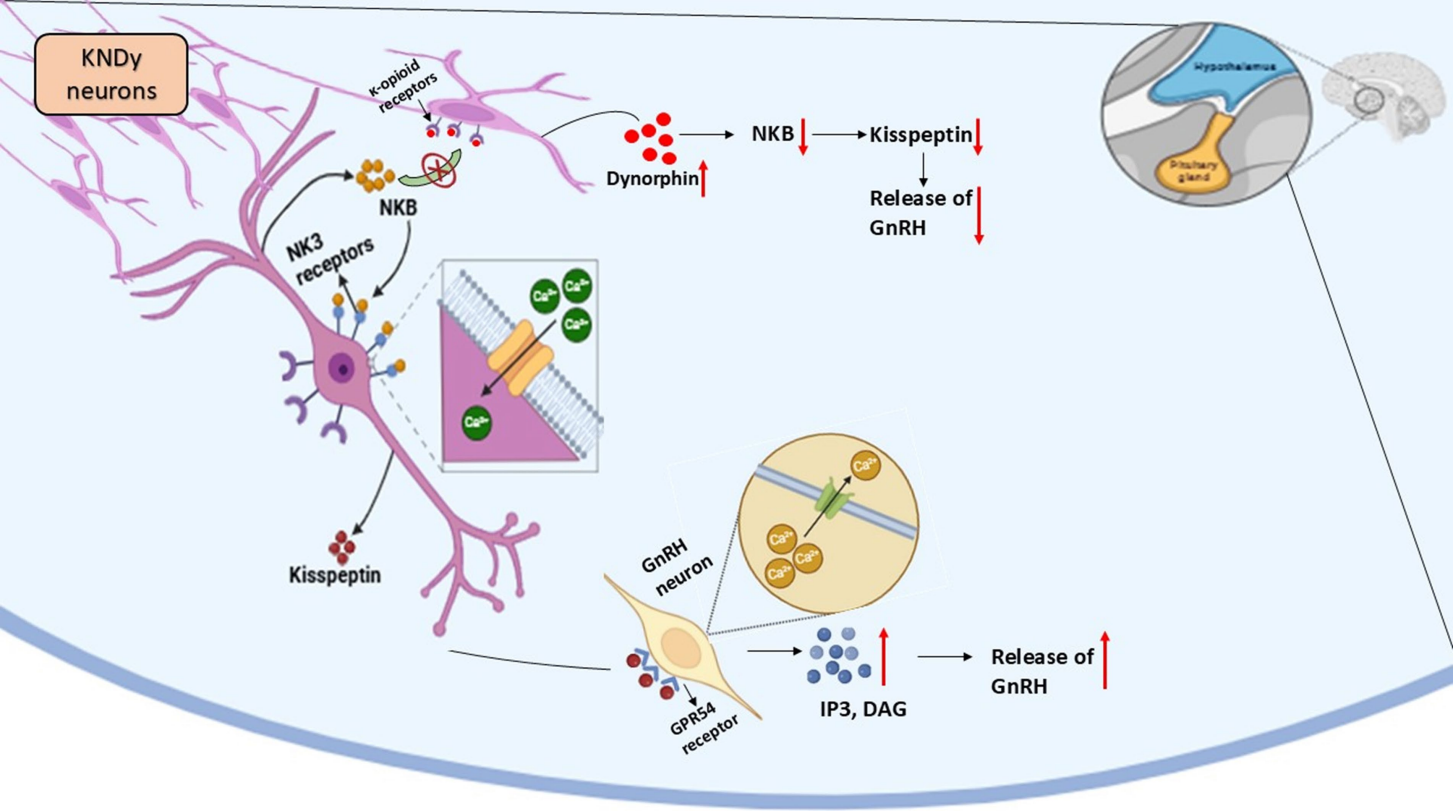
Kisspeptin-neurokinin B-Dynorphin (KNDy) neuronal regulation of reproduction in seasonal breeders.

- The three main regulators that control KNDy neuron function are kisspeptin, together with NKB and Dyn, but SP (Substance P) and NKA (Neurokinin A) may also contribute to this modulation (77).
- Additional research about alternative regulatory mechanisms of GnRH secretion that bypass kisspeptin pathways should be conducted (76) (Figure 3).

The summary of the molecular pathway is shown in Table 3.

**Table 3:** Kisspeptin-neurokinin B-Dynorphin (KNDy) neuron regulation in seasonal breeders.

Factor	Effects on reproductive functions
Function of KNDy neurons	Located in the arcuate nucleus (ARC), co-expresses Kisspeptin, Neurokinin B (NKB), and Dynorphin (Dyn) to regulate GnRH secretion (68).
GnRH Pulsatility regulation	NKB stimulates KNDy neurons → Increases Kisspeptin release → Activates GnRH neurons. Dynorphin provides negative feedback to regulate GnRH pulses (173).
Estrogen feedback	KNDy neurons mediate estrogen’s positive and negative feedback on GnRH secretion, maintaining reproductive balance (72).
Experimental evidence	NK3-SAP ablation of KNDy neurons eliminates GnRH pulses. Loss of KNDy neurons leads to reduced gonadotropin release, infertility, and metabolic changes (174).
Role in seasonal breeders	Shorter daylight → Increased melatonin → Suppresses KNDy activity → Reduced GnRH and reproductive quiescence. Longer daylight → Decreased melatonin → Reactivates KNDy neurons → Restores fertility (76).
Additional regulatory factors	Substance P (SP) and Neurokinin A (NKA) also contribute to KNDy neuron modulation, especially in response to environmental cues like photoperiod (175).
Future research directions	Explore the precise mechanisms by which KNDy neurons integrate photoperiodic and metabolic cues to regulate GnRH pulsatility to modulate reproductive timing and enhance fertility during non-breeding seasons (176).

## AMPK-MTOR energy sensing pathway (nutritional effect on reproduction)

Organisms need high amounts of energy to produce offspring, and metabolic conditions control reproductive regulatory mechanisms. The AMP-activated protein kinase (AMPK), together with the mammalian target of rapamycin (mTOR), constitutes important cellular energy sensors regulate reproductive function by sensing metabolic signals (78).

### AMPK: the energy sensor in reproductive regulation

#### Activation and function

- The enzyme AMPK starts its operation when energy supplies fall low (such as during fasting or periods of caloric restriction) to save fuel (79).
- The energy-saving process includes steroidogenesis, follicular development, and ovulation, which AMPK prevents during times of low energy (80).
- The activation of AMPK leads to the prevention of GnRH release, which results in delayed puberty and deficient reproductive function (78).

#### AMPK in follicular development

- Healthy Ovarian cells, together with oocytes and theca cells, express high levels of AMPK protein (81).
- The maturation of oocytes becomes delayed through AMPK activation because it blocks signaling pathways required for meiosis (82).
- Research findings demonstrate that blocking AMPK activity helps follicles grow, which implies that activated AMPK controls reproductive function as an energy deficit regulator (83).

#### AMPK in granulosa cell function and hormone secretion

- Under FSH and IGF-I regulation, granulosa cells create both estrogen and progesterone compounds (84).
- Through activation of AMPK, the production of progesterone decreases by preventing the function of steroidogenic acute regulatory (StAR) protein and 3β-hydroxysteroid dehydrogenase (3β-HSD) (78).
- The activation of AMPK by metformin treatment results in suppressed granulosa cell proliferation in ruminant cattle, which leads to modifications in follicular development (85).

#### mTOR: the energy sensor for reproductive activation

Under high-energy conditions, mTOR acts as a crucial controller of reproductive function while managing cell growth together with protein synthesis and reproductive abilities (86). The activation of mTOR leads to cellular growth, protein synthesis, and reproductive functions through stimulation of follicular development, steroid hormone production, and oocyte maturation. The ovarian system requires mTOR activation to activate primordial follicles and stimulate granulosa cell growth, together with ovulation (87). The medication rapamycin, together with other mTOR inhibitors, blocks follicular development, which can result in infertility. The research on seasonal breeders has established that mTOR signaling decreases in periods outside breeding seasons, which results in reproductive dormancy (88).

Reproductive function requires the oppositional regulatory mechanism between mTOR and AMPK, which interact with each other (89). Under situations of energy deficiency, AMPK becomes active, thus it blocks mTOR signaling to reduce reproductive processes for metabolic energy conservation (90). The reproductive process gets activated through mTOR signaling, while energy-rich conditions lead to AMPK suppression. The AMPK, together with mTOR, works in a balanced opposition to maintain reproductive outcomes based on metabolic health status (91).

Essential for determining reproductive cycles in donkeys and other seasonal breeding species is the metabolic regulation mechanism (78). Low food availability leads to AMPK activation, which inhibits GnRH secretion along with reproductive functions, thus stopping the expenditure of energy for reproduction. An increase in food availability results in mTOR activation, which leads to ovarian function, thereby allowing reproduction to occur only in favorable metabolic situations. Seasonal breeding creatures use nutrition-dependent reproductive regulation to maximize their reproductive performance (92).

**Figure 4:**
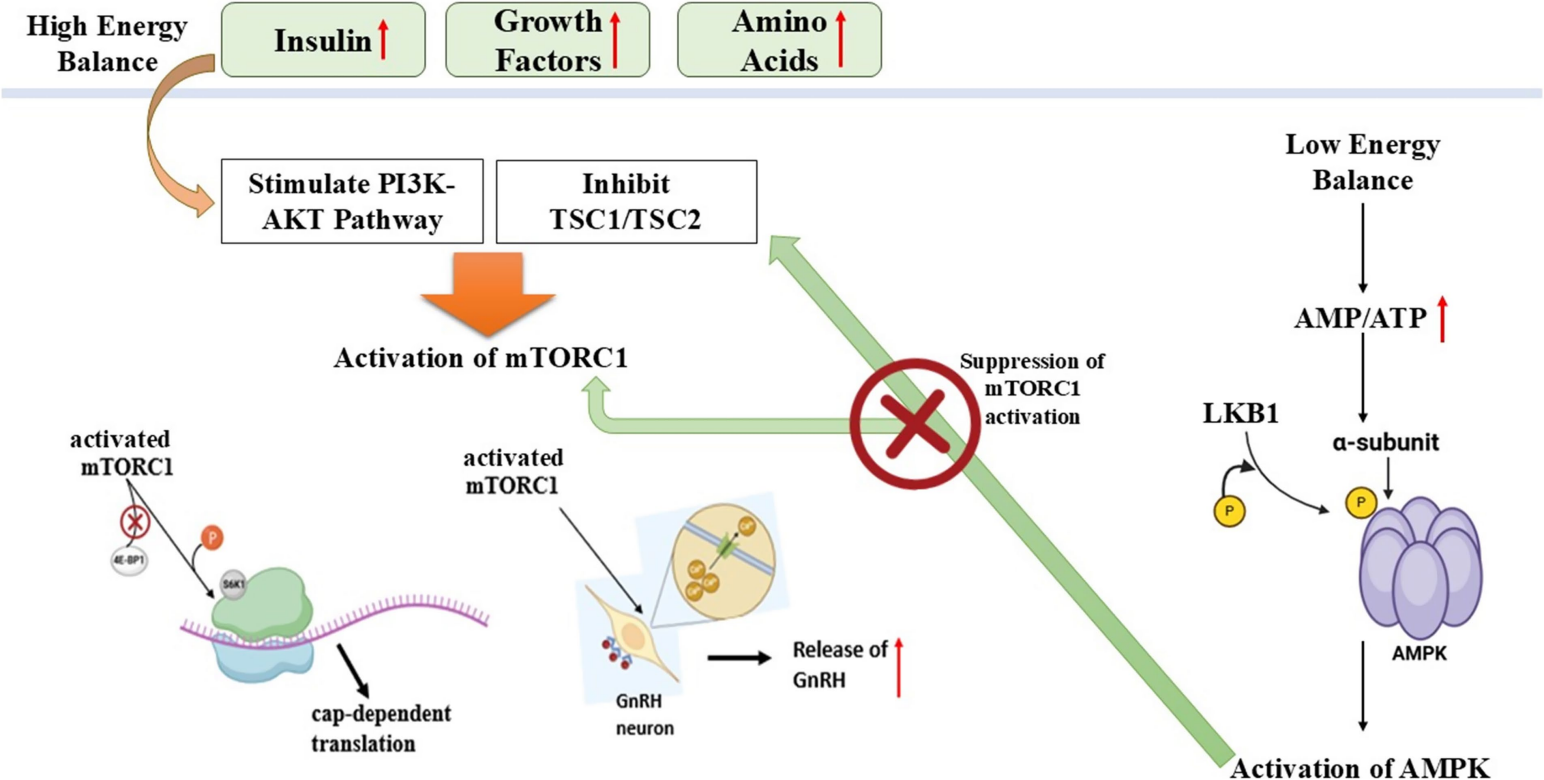
Schematic illustration of AMPK-MTOR energy sensing pathway regulating reproduction in seasonal breeders (Nutritional effect on reproduction).

The KNDy neuron system together with the AMPK-mTOR pathway acts as an important regulatory mechanism for reproductive efficiency in seasonal breeding animals (93). GnRH pulsatility depends on signals from KNDy neurons, which receive photoperiod information through melatonin signaling along with the AMPK-mTOR pathway acting as a metabolic control mechanism for reproduction under sufficient energy conditions. Knowledge about these pathways reveals crucial information about seasonal reproductive control, thus offering possibilities to develop fertility enhancement practices for domestic animals (79). The summary of the molecular pathway is shown in Table 4 (Figure 4).

**Table 4:** AMPK-mTOR energy sensing pathway and its role in reproductive regulation.

Factor	Effects on reproductive functions
Role of AMPK in energy sensing	Activated under low-energy conditions (fasting, caloric restriction) to conserve metabolic resources by suppressing reproduction (177).
AMPK and reproductive inhibition	Prevents steroidogenesis, follicular development, and ovulation. Suppresses GnRH release, delaying puberty and reducing reproductive function (78).
AMPK in follicular development	Highly expressed in ovarian cells, oocytes, and theca cells. Delays oocyte maturation by inhibiting meiosis-related signaling pathways. Blocking AMPK activity promotes follicular growth (178).
AMPK in granulosa cell function	Regulates estrogen and progesterone production under FSH and IGF-I control. Inhibits StAR protein and 3β-HSD, reducing progesterone levels. Metformin-induced AMPK activation suppresses granulosa cell proliferation in ruminants, altering follicular development (78).
Role of mTOR in reproductive activation	Activated under high-energy conditions to promote cell growth, protein synthesis, and reproductive functions. Stimulates follicular development, steroidogenesis, and oocyte maturation. Essential for primordial follicle activation and granulosa cell proliferation (87).
mTOR and seasonal reproductive dormancy	mTOR signaling decreases outside breeding seasons, leading to reproductive dormancy. Rapamycin and other mTOR inhibitors suppress follicular development, potentially causing infertility (179).
AMPK-mTOR interaction	AMPK inhibits mTOR under energy-deficient conditions to prevent reproductive activation. mTOR suppresses AMPK under energy-rich conditions to promote reproductive functions (180).
Nutritional regulation of reproduction	Low food availability activates AMPK, inhibiting GnRH and reproductive processes to conserve energy. High food availability activates mTOR, restoring ovarian function and reproductive activity (91).
Integration with seasonal breeding	The AMPK-mTOR pathway interacts with KNDy neurons and melatonin signaling to regulate reproductive cycles. Understanding these mechanisms aids in fertility management strategies for domestic seasonal breeders like donkeys (181).

#### Ovarian transcriptomic profiles of donkeys

Transcriptomic study of donkey granulosa cells has demonstrated high enrichment of PI3K-Akt and focal adhesion pathways, suggesting active participation in cell proliferation, steroidogenesis, and follicular support (32). Differential expression of genes, e.g., endomucin (EMCN) and synaptotagmin-like protein 12 (SYT12), indicates potential molecular actors that are specific to donkey follicular biology (94). Sheep follicular gene networks are well described, and horse research is also growing, but donkeys are poorly characterized at the transcriptomic level (95).

## Steroidogenesis pathway disruption

The reproductive efficiency of seasonal breeders, including donkeys, is regulated by ovarian steroidogenesis through its vital function. The reproductive cycles depend on correct sex hormone synthesis that results from this process to support follicular development and regulate ovulation (96). The pathway of ovarian steroidogenesis undergoes disruption when exposed to endocrine-disrupting chemicals (EDCs) since these chemicals create hormonal imbalances that negatively affect fertility processes (97). The function of sex hormones and hormone receptors becomes disrupted because of environmental chemicals, which are mainly present in pesticides, plastics, and industrial waste, thus resulting in reproductive complications. The evaluation of seasonal breeders requires knowledge about how EDCs modify steroidogenesis at the molecular level (98).

### Ovarian steroidogenesis and its regulation

The ovary produces sex hormones through a coordinated process involving two different cell types as well as two different hormones (99). Luteinizing hormone (LH) activates cholesterol conversion to androgens in the theca cells so that these hormones move on to granulosa cells. The hormone FSH in granulosa cells turns on aromatase activity that transforms androgens into estradiol (100). The restrictive hormone control system completes the proper functioning of estrous cycles and ovulation while sustaining pregnancy in seasonal reproduction cycles. Hormone production becomes impaired through disruptions in this pathway, which occurs from environmental stressors or EDC exposure, thus causing irregular reproductive cycles along with infertility (101).

### Endocrine disrupting chemicals interference in ovarian steroidogenesis

EDCs interrupt ovarian steroidogenesis either by blocking essential enzymes, duplicating natural hormones, or obstructing various receptors (97). Studies show that the chemical substances bisphenol A (BPA), phthalates, and Polychlorinated Biphenyls (PCBs) block aromatase activity, which decreases estradiol production. Pesticides together with dioxins disrupt the steroidogenic acute regulatory (StAR) protein required for cholesterol transport into mitochondria (102). These environmental toxins interfere with vital molecular pathways to change the regulation of the estrous cycle as well as the reproductive efficiency of species that align with seasonal mating patterns (103).

### Impact on reproductive function in seasonal breeders

The reproduction of seasonal breeders such as donkeys strongly depends on environmental clues, including photoperiod and nutrition, because disruptions in steroidogenesis cause major reproductive effects (104). Endocrine-disrupting compounds affecting sex hormone equilibrium control the duration of the estrous cycle, delay ovulation, and decrease fertility potential (105). Juvenile animals become unable to sustain pregnancy because the corpus luteum function fails to maintain normal progesterone levels, and when estrogen production becomes disrupted, it affects follicle maturation. The dependence of these species on hormonal changes for seasonal reproduction makes exposure to EDCs a possible cause of declining fertility and reproductive problems (106).

**Figure 5:**
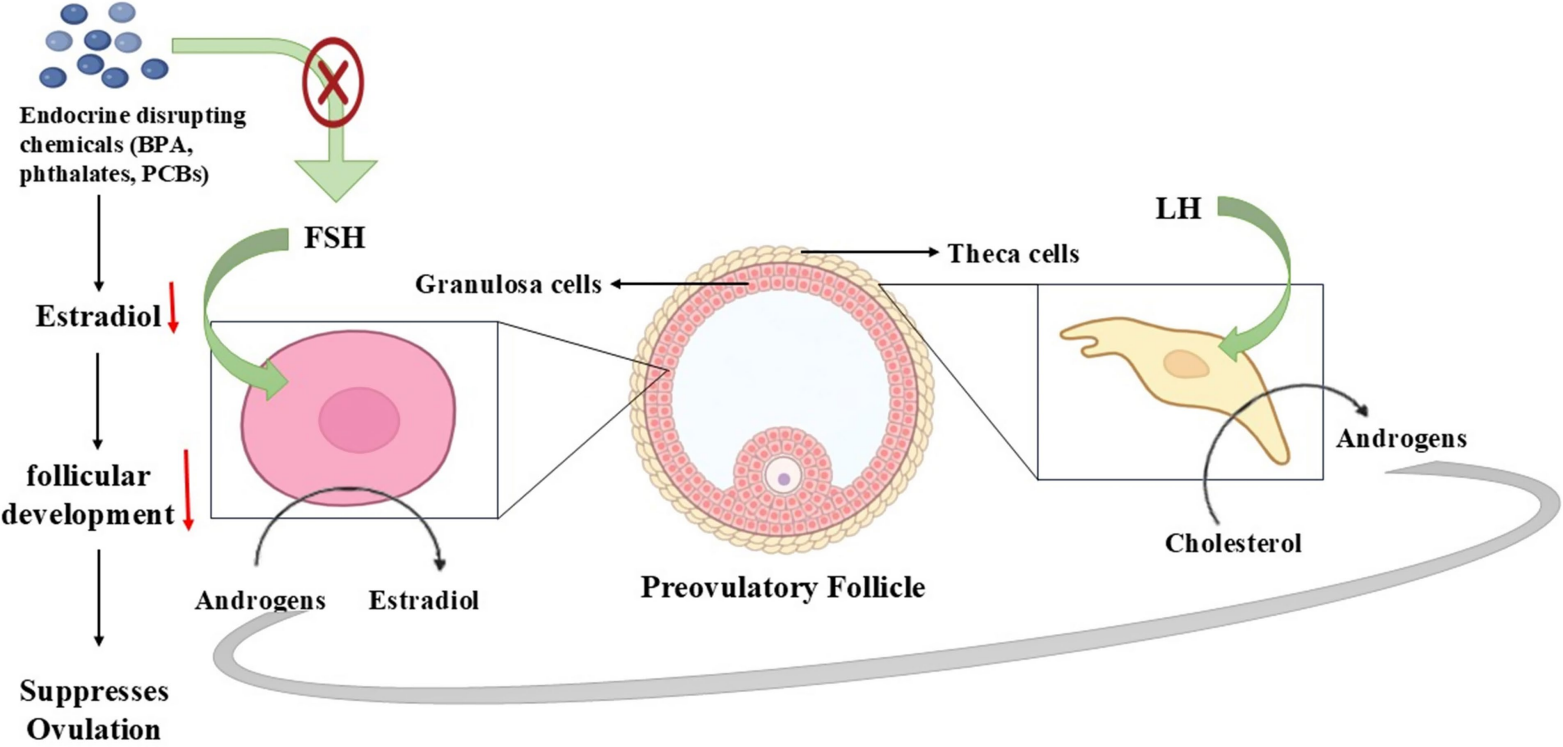
Steroidogenesis pathway disruption affecting reproduction in seasonal breeders.

The essential hormonal process of steroidogenesis controls reproductive efficiency, but environmental pollutants create major difficulties for seasonal breeders to maintain their reproductive functions (107). The impact of environmental pollutants on hormone biosynthesis steps results in reproductive breakdowns, which creates permanent effects on fertility (108). Research must be conducted to determine how much seasonal breeders encounter environmental pollutants while developing new approaches to lessen the reproductive health damage. The identification of environmental polluting factors will enable better decision-making regarding protection plans for species suffering from pollution exposure effects (109) (Figure 5).

The summary of the molecular pathway is shown in Table 5.

**Table 5:** Steroidogenesis pathway disruption and its impact on seasonal breeders.

Aspect	Details
Role of steroidogenesis in reproduction	Ovarian steroidogenesis is essential for reproductive efficiency in seasonal breeders, including donkeys. Regulates sex hormone synthesis, follicular development, ovulation, and pregnancy maintenance (182).
Endocrine-disrupting chemicals (EDCs) and their effects	EDCs cause hormonal imbalances, leading to reproductive dysfunction. Found in pesticides, plastics, industrial waste, and environmental pollutants (183).
Mechanisms of EDC interference in steroidogenesis	EDCs block essential steroidogenic enzymes, mimic natural hormones, or disrupt hormone receptors. BPA, phthalates, and PCBs inhibit aromatase, reducing estradiol production. Pesticides and dioxins impair StAR protein function, blocking cholesterol transport into mitochondria (184).
Impact on seasonal breeders	Alters estrous cycle duration and ovulation timing. Disrupts corpus luteum function, leading to inadequate progesterone levels. Affects follicle maturation and reduces fertility potential (185).
Consequences of steroidogenesis disruption	Reproductive inefficiency and fertility decline in seasonal breeders. Increased pregnancy loss due to hormonal imbalances. Long-term environmental exposure may permanently impact reproductive health (97).
Future research needs	Investigate the extent of EDC exposure in seasonal breeders. Develop strategies to mitigate environmental pollutant effects on reproduction. Identify protective measures to enhance fertility in species affected by pollution (98).

## Oxidative stress pathways and apoptosis in gonads

The reproductive performance of seasonal breeders, including donkeys, depends significantly on oxidative stress (OS) because their reproductive cycles follow environmental signals closely (110). The correct relationship between reactive oxygen species (ROS) and antioxidants is necessary for proper reproductive system operation (111). When ROS production becomes excessive, it interferes with the balance, which subsequently damages cells through steroidal hormone production failure and triggers cell death in gonadal tissue (112). The reproductive functions of oocyte maturation and sperm function, together with embryonic development, undergo disturbances that affect fertility and seasonal breeding performance (113).

### Oxidative stress in reproductive tissues

The metabolism of cells produces ROS byproducts mainly in mitochondria, which serve as crucial signaling agents during folliculogenesis and ovulation and corpus luteum development (114). High levels of ROS exceed the capacity of antioxidants to control them, which results in damage to lipids, proteins, and DNA, leading to gonadal cell death through apoptosis (115). The reproductive patterns of seasonal breeders respond directly to photoperiodic changes and metabolic status, thus making this research important for their breeding cycles (116). The level of oxidative stress tends to increase throughout the non-breeding cycle to maintain reproductive dormancy, yet specific ROS regulation helps execute important reproductive processes, including follicular rupture together with sperm capacitation during the breeding period (117).

### Apoptosis in gonads and its regulation

Gonadal functionality depends on programmed cell death known as apoptosis since this process regulates the death of follicles while also controlling sperm formation (118). Seasonal breeders primarily depend on the intrinsic apoptotic pathway, which originates from mitochondrial dysfunction combined with oxidative damage to their cells (119). Excessive ROS activates cytochrome c release from mitochondria to activate caspases, which in turn causes the death of follicular cells and germ cells (120). The existence of a balance between pro-apoptotic proteins BAX and BAK and anti-apoptotic protein BCL-2 decides whether cells will survive. The reproductive efficiency of reproductive systems is impacted by season-dependent modifications of gonadotropin levels and melatonin signaling that control oxidative stress mechanisms and apoptosis rates in gonadal tissues (121).

### Impact on reproductive efficiency in seasonal breeders

The reproductive efficiency of seasonal breeders such as donkeys is directly affected by oxidative stress and apoptosis because they harm gametes and their reproductive organs’ functionality (122). Controlled oxidative signaling supports ovulation together with sperm maturation during the breeding season. Excessive oxidative damage during times outside the breeding period quickens the process of follicular atresia while causing sperm viability to decrease (123). Reproductive success suffers from environmental stressors such as heat exposure, poor nutrition, and toxic environmental substances, which increase the rate of oxidative damage in animals (124).

### Strategies to mitigate oxidative stress

The improvement of reproductive performance in seasonal breeders depends on implementing methods that reduce oxidative stress damage (125). The combination of antioxidant supplements, including vitamins C and E, and selenium, and melatonin, leads to better gonadal function and fertility results (126). The reproductive potential can benefit from nutritional measures that activate endogenous antioxidant enzymes, including superoxide dismutase, catalase, and glutathione peroxidase (127). Proper control of environmental stressors together with appropriate nutritional provision during breeding seasons will help reduce oxidative damage, which in turn leads to improved reproductive outcomes across donkeys and other seasonal breeders (128).

**Figure 6:**
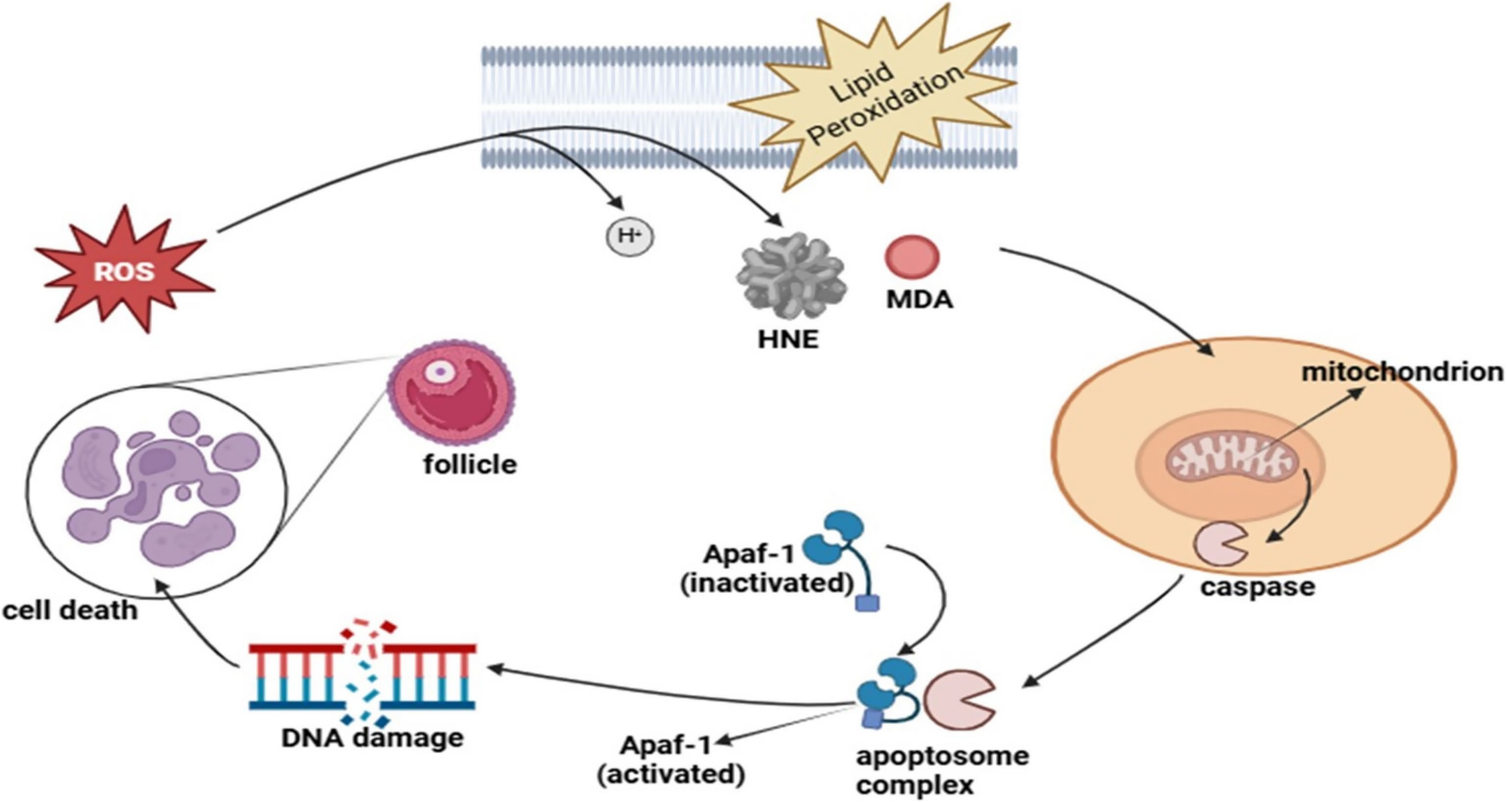
Role of oxidative stress pathways and apoptosis in gonads in the regulation of reproduction in seasonal breeders.

The regulation of reproductive efficiency in seasonal breeders depends heavily on oxidative stress together with apoptosis mechanisms (129). The body needs regulated ROS production to maintain normal reproductive functions, yet too much oxidative damage triggers problems with gamete quality along with hormonal imbalancing and infertility (130). Laboratory research on oxidative stress mechanisms interacting with seasonal reproductive signals will enable scientists to develop better treatments for enhancing donkey breeding performance alongside other seasonal breeders (131) (Figure 6).

The summary of the molecular pathway is shown in Table 6.

**Table 6:** Oxidative stress pathways and apoptosis in gonads of seasonal breeders.

Aspect	Details
Role of oxidative stress (OS) in reproduction	Seasonal breeders, including donkeys, rely on a balance between reactive oxygen species (ROS) and antioxidants. Excessive ROS disrupts reproductive functions, affecting oocyte maturation, sperm function, and embryonic development (115).
Sources and effects of ROS in gonadal tissues	ROS are byproducts of mitochondrial metabolism, playing key roles in folliculogenesis, ovulation, and corpus luteum function. Uncontrolled ROS levels lead to lipid peroxidation, protein oxidation, DNA damage, and gonadal cell apoptosis (186).
Seasonal variation in oxidative stress	OS increases during the non-breeding season to maintain reproductive dormancy. Regulated ROS levels are necessary for follicular rupture and sperm capacitation during the breeding season (22).
Apoptosis in gonads and its regulation	Apoptosis controls follicular atresia and spermatogenesis. Mitochondrial dysfunction due to oxidative stress triggers cytochrome c release, activating caspases for cell death. The balance between pro-apoptotic proteins (BAX, BAK) and anti-apoptotic proteins (BCL-2) determines cell survival (118).
Impact on reproductive efficiency	Excess ROS during non-breeding periods accelerates follicular atresia and reduces sperm viability. Environmental stressors (heat, poor nutrition, toxins) exacerbate oxidative damage, impairing fertility (187).
Strategies to mitigate oxidative stress	Antioxidant Supplementation: Vitamins C, E, selenium, and melatonin improve gonadal function and fertility. Nutritional Interventions: Activation of endogenous antioxidant enzymes (SOD, catalase, glutathione peroxidase). Environmental Management: Reducing stressors and optimizing nutrition during the breeding season (188).
Future research needs	Need to focus on identifying reliable oxidative biomarkers and evaluating antioxidant-based therapeutic strategies that could enhance fertility regulation across reproductive seasons (115).

## Prolactin pathway in seasonal breeders

As a key hormone in seasonally breeding animals, prolactin (PRL) regulates reproductive periods and helps the body adapt to environmental shifts (132). The hormone shows seasonal patterns where the secretion rate reaches its highest point during the spring and summer months and its lowest point in the autumn and winter months (133). The hormonal regulation of prolactin depends mostly on Photoperiod which controls pineal gland melatonin production and ultimately directs prolactin output (134).

### Photoperiodic regulation of prolactin

The duration of daylight throughout seasons strongly controls the production of melatonin and therefore controls the anterior pituitary’s release of prolactin (135). The hypothalamus, along with the pars tuberalis of the pituitary, contains receptors that allow melatonin to trigger seasonal endocrine responses (136). The control mechanism for both gonadotropins as well as prolactin functions through the shared regulation of luteinizing hormone (LH), follicle-stimulating hormone (FSH), and prolactin by melatonin (137).

The mechanisms that regulate seasonal prolactin changes differ from gonadotropin patterns because prolactin relies on direct neuroendocrine regulation, but gonadotropins follow feedback-based control (138). Current research does not provide enough evidence to prove that winter prolactin reduction happens only through increased dopamine inhibition (139). At this time, pituitary becomes more responsive to dopamine, which could be a factor in the decrease of prolactin, secretion throughout seasonal cycles. The intricate relationship between melatonin, prolactin and gonadotropins demonstrates how the human body readjusts reproductive and metabolic systems because of seasonal variations (132).

### Prolactin’s role in seasonal reproduction

- Certain species rely on prolactin as their luteotrophic factor for corpus luteum maintenance during pregnancy (140).
- The activity of prolactin as an implantation delay factor affects the timing of embryo attachment in Bennett’s wallaby, along with the tammar wallaby (141).
- The seasonal prolactin secretion pattern seems to be a fundamental biological trait that affects reproductive cycles as well as fur shedding (molt) and bodily metabolism (142).

### Prolactin and pituitary interactions in seasonal breeders

- The pituitary gland contains prolactin receptor proteins inside both the pars distalis and pars tuberalis areas indicating a paracrine regulatory internal process (132).
- Gonadotropes (LH and FSH-secreting cells) and lactotropes (PRL-secreting cells) demonstrate direct physical contact throughout the pituitary, but their structural relationships fluctuate between seasons according to research findings (134).
- The hormone dopamine functions to block prolactin release while some species demonstrate that prolactin maintains control over how gonadotropes react to GnRH which helps stop the glands from overstimulation (143).
- The photoperiod of the breeding environment determines how powerfully prolactin inhibits gonadotropin secretion through its photoperiodic dependency, where short-day breeders (sheep) demonstrate stronger inhibition but long-day breeders (horses) display a more regulatory effect (144).

### Molecular and cellular mechanisms

The regulatory patterns of pituitary hormone secretion might be influenced by seasonal shifts observed in folliculostellate cells, which belong to the category of pituitary support cells (145). Research indicates that breeding_season triggers an increase in cell-adherens junctions between these cells while prolactin and gonadotropin interactions simultaneously evolve (146). The GnRH hormone canstimulate prolactin release, but its effect on this process depends on the season, the species, and the reproductive condition of the animal (147).

**Figure 7:**
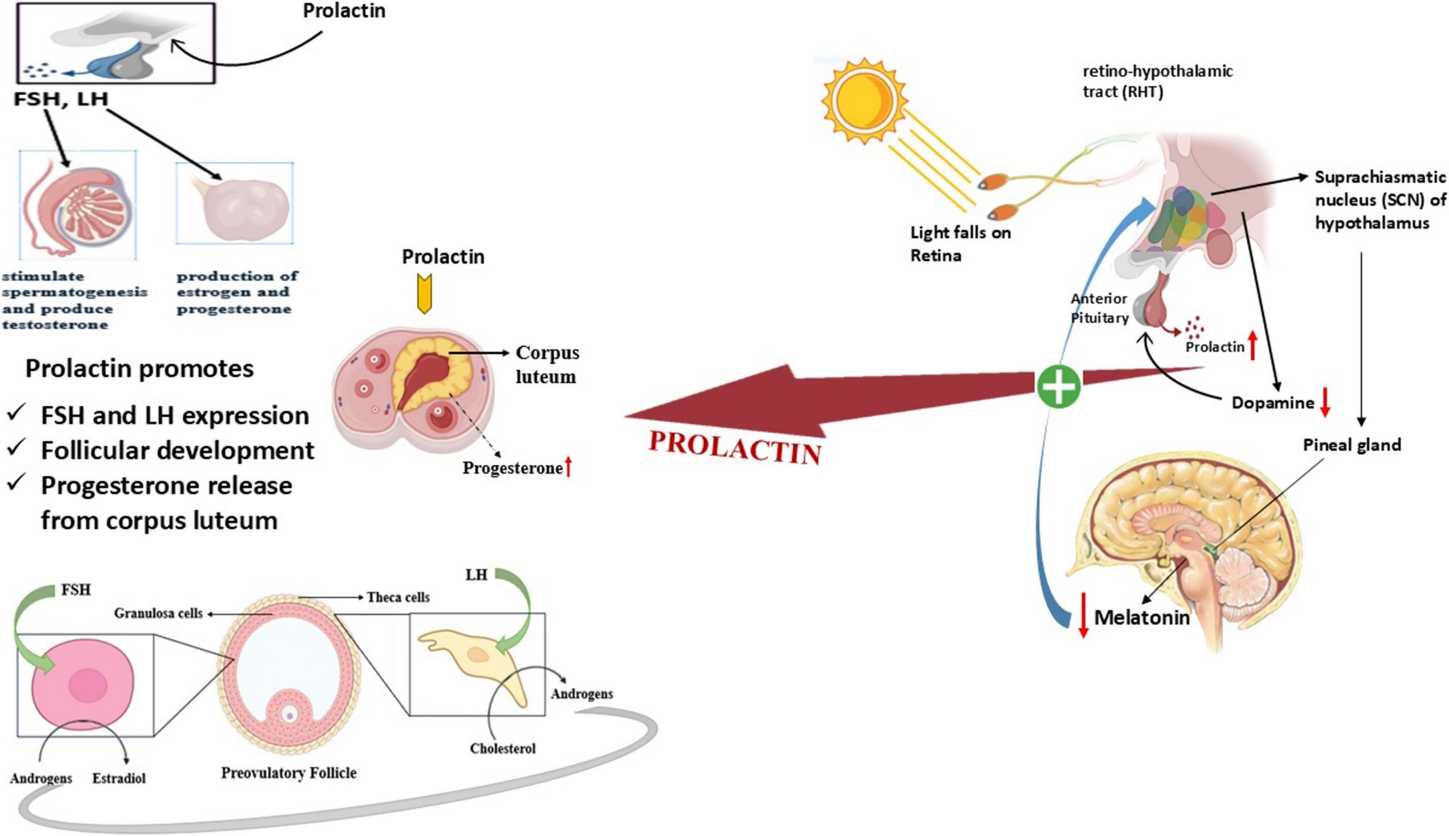
Role of prolactin pathway in the regulation of reproduction in seasonal breeders.

Many mammals use prolactin as their main seasonal regulatory agent while photoperiodic cues processed through melatonin pathways control its secretions (148). Prolactin plays an essential part in seasonal physiological adaptations since it controls molt, metabolism and energy balance beyond reproduction (149). The complex dynamic system of prolactin together with gonadotropins and hypothalamic regulatory components shows that it plays an essential role in reproductive adaptation to environmental changes while managing energy, and reproductive resource distribution annually (150) (Figure 7).

The summary of the molecular pathway is shown in Table 7.

**Table 7:** Prolactin pathway in seasonal breeders.

Aspect	Details
Role of prolactin (PRL) in seasonal breeding	PRL regulates reproductive cycles in response to environmental changes. It peaks in spring/summer; lowest levels in autumn/winter, and is controlled by photoperiod via melatonin signaling (132).
Photoperiodic regulation of PRL	Daylight duration influences melatonin release, which affects PRL secretion. Melatonin receptors in the hypothalamus and pars tuberalis mediate seasonal endocrine responses. PRL is regulated neuroendocrinally, while gonadotropins follow feedback-based control (133).
Prolactin and pituitary interactions	PRL receptors are present in the pars distalis and pars tuberalis of the pituitary. Gonadotropes (LH/FSH cells) and lactotrophes (PRL-secreting cells) interact seasonally. Dopamine inhibits PRL release; PRL modulates gonadotrope responsiveness to GnRH. Short-day breeders (e.g., sheep) show stronger PRL inhibition on gonadotropins than long-day breeders (e.g., horses) (189).
Molecular and cellular mechanisms	Seasonal changes influence folliculostellate cells in the pituitary, affecting hormone secretion. GnRH can stimulate PRL secretion depending on species and reproductive status (132).
Research implications	Further studies on investigating its role in follicular development, luteal maintenance, and interaction with dopamine and GnRH pathways to optimize breeding outcomes during the off-season (133).

Thus, the prolactin pathway serves as a vital neuroendocrine mechanism in seasonal breeders, regulating reproductive timing and physiological adjustments in response to the photoperiod (133). The regulation of prolactin secretion in response to melatonin affects both the reproductive and metabolic functions (132). The pituitary paracrine interactions and dopamine modulation indicate the association between prolactin and gonadotropin output (151). Thus, it indicates the dual role of prolactin in regulating internal hormonal rhythms and external seasonal changes.

The molecular pathways coordinate in response to photoperiod and nutritional availability, ensuring optimal reproduction during long days (151). The summary of these molecular pathways is shown in Table 8. It is well established that the reproductive activity in donkeys is regulated by interconnected hormonal, signaling, and metabolic pathways that respond to the environmental and seasonal variability (16). Thus, disruptions in the HPG axis, melatonin signaling, KNDy neurons, and prolactin pathway result in decreased production of GnRH and gonadocorticoids (10). Malnutrition also affects the AMPK-mTOR pathway, resulting in declined reproductive performance, and oxidative stress leads to gonadal cell damage. Understanding these mechanisms broadens our knowledge of seasonal fertility and opens the horizon for improving reproductive efficiency through modulation of molecular pathways using hormonal, nutritional, and management strategies in seasonal breeders, particularly in donkeys.

**Table 8:** Summary of the molecular pathways involved in seasonal reproductive decline.

Pathway	Key molecules	Effect on reproduction
HPG Axis dysregulation	GnRH, LH, FSH, Testosterone, Estradiol (21)	↓ HPG function reduces fertility (190)
Melatonin pathway	Melatonin (MT1, MT2), Kisspeptin, NKB (169)	↑ Melatonin inhibits GnRH secretion (191)
KNDy neurons	Kisspeptin, NKB, Dynorphin (76)	↓ Kisspeptin → ↓ GnRH release (75)
AMPK-mTOR energy sensing	AMPK, mTOR, Leptin (78)	Poor nutrition suppresses reproduction (192)
Steroidogenesis disruption	StAR, CYP17A1, 3β-HSD, 17β-HSD (182)	↓ Testosterone, Estradiol synthesis (193)
Oxidative stress and apoptosis	ROS, SOD, BAX/Bcl-2[136]	↑ Gonadal cell death reduces fertility (115)
Prolactin pathway	Prolactin, dopamine (133)	↑ Prolactin suppresses GnRH and steroidogenesis (132)

### Scope of ART in donkey

Thus, donkeys have seasonally regulated reproductive patterns which are mainly regulated by photoperiod (152). Reproductive traits of males (e.g., testicular size, semen quality, hormonal changes (e.g., testosterone)) differ in breeding and non-breeding seasons (153). The results of the immunohistochemical studies of the epididymis indicate the presence of higher epithelial activity and sperm in the spring, whereas higher markers of oxidative stress and autophagy are observed in off-seasons (154). These results deny the previous hypotheses of low seasonality and support the importance of time-adjusted breeding plans. In addition, Dezhou donkeys immunized against inhibin exhibited elevated levels of FSH, LH, testosterone, and activin A, especially out of breeding season (155).

Donkeys and horses are very different in their reproductive behavior, the duration of a cycle, and anatomical characteristics (27). The estrous cycle and the gestation period of Jennies are longer, and they also respond to factors other than photoperiod (12). Jacks have bigger reproductive organs, and they take more time to ejaculate (152). Behavioral peculiarities of the donkey reproduction, like territoriality and non-harem mating patterns, as well as reduced spermatogenic efficiency, also distinguish it among other equids (27). These characteristics require specific assisted reproductive technology (ART) regimens. It has been found that duration of the follicular phase, not luteolysis, is the main factor determining variability in interovulatory interval (IOI) (156). Longer IOIs are associated with longer estrus, slower follicle development, and larger follicles at ovulation. Such findings are important to schedule insemination and forecast fertility periods (27).

Thc sperm of donkeys has some cryobiological difficulties. The traditional freezing techniques produce uneven fertility outcomes, particularly in jennies (157). Nevertheless, the latest developments in sperm vitrification show positive results. Straws with outer covers, using 0.25 mL straws, showed similar or better motility and *in vivo* fertility than standard frozen semen (158). Remarkably, vitrified semen caused a less severe and short-term uterine inflammatory reaction (159). Moreover, Phospholipase C Zeta (PLCzeta) localization in donkey sperm showed that it was competent in oocyte activation, particularly during intracytoplasmic sperm injection (ICSI) into horse oocytes (160). These interspecies ICSI outcomes confirm the utilization of donkey sperm in the creation of mules and imply the expansion of ARTs (161).

Endometritis is a significant limitation to fertility in donkeys. The use of equine-based histopathological grading of donkey uteri has been effective, and cytological and biopsy-based assessment would improve the level of diagnosis (162). These aids enable a superior categorization of uterine health and an even more accurate treatment regimen. Donkeys are prone to metabolic problems that affect reproduction. Metabolic disorders like insulin dysregulation, hyperlipemia, and Pituitary Pars Intermedia Dysfunction (PPID) are usually compounded by obesity (29). These conditions either directly or through systemic effects lead to impaired reproductive performance (e.g., laminitis, organ dysfunction). Most unfortunately, the majority of hormonal reference ranges and treatment protocols are based on horses, in spite of pharmacokinetic differences (163).

Breed size also plays a great role in reproductive performance. Big-bodied breeds such as the Dezhou donkey are more fertile and produce more milk than smaller breeds (e.g., Cullen donkeys) (164). Surveys conducted in Northern China indicate that formalized farm activities, especially with national/provincial institutions, are associated with improved ART adoption and reproductive success (153). The survey shows that about 73 percent of the surveyed farms are using artificial insemination, indicating the rising use of ART. Such results support the importance of breed selection, genetic advancement, and farm standardization in optimizing fertility. Donkeys are increasingly being used in protecting endangered equids. ARTs have advanced in horses, but adaptation to donkeys and wild equids is continuing (12). Donkeys can be used as fertility models as well as surrogates in conservation programs, particularly due to their reproductive strength and availability (165). Nevertheless, the molecular variability of gamete behavior and endocrine response requires specific studies. The donkey ARTs should be aligned with the principles of conservation biology in order to save genetic diversity in Equus (159).

### Research gaps and future directions

Although there has been an improvement, there are still considerable gaps in our knowledge of donkey reproductive physiology and molecular control. The principal areas in need of focus are:

- Creation of hormonal and metabolic reference values in donkeys
- Pharmacological validation of this species
- Explanation of molecular mechanisms of seasonal modulation of fertility
- Improving ART procedures such as ICSI and embryo transfer
- Reproductive studies of donkey, horse, and mule to compare to find out some unique limitations and possibilities

To resolve these problems, interdisciplinary cooperation, the use of sophisticated molecular technologies, and the dedication to the species-specific research framework will be needed.

## Conclusion

The multifactorial nature of the reproductive inefficiency in donkeys has its basis in the underrecognized physiological peculiarity and the lack of specific molecular studies. This review offers convincing details of photoperiod-induced seasonal fertility, metabolic endocrine imbalances, and anatomical differences that determine reproductive fitness. New opportunities are available with recent advances in ARTs, endocrinology, and histological profiling as ways to improve fertility and conservation results. It is now necessary to strategically invest in donkey-specific research and comparative reproductive biology to deliver these insights into productive breeding innovations and long-term species sustainability. In addition, the combination of management practices with molecular insights can increase reproductive efficiency, improve animal welfare, and increase the productivity of the donkey populations. Such integration of scientific basic research and applied animal husbandry will be essential to overcoming the limitations imposed by seasonal infertility and maximizing reproductive performance in this important but often neglected species. So, to boost farm production efficiency of seasonal breeders like donkeys, upgrading reproductive efficiency by adopting cutting-edge animal biotechnological tools and breeding technologies is the future of donkey farming.
